# Editorial: Induction of Central Nervous System Disease by the Adaptive Immune Response

**DOI:** 10.3389/fimmu.2017.01218

**Published:** 2017-10-02

**Authors:** Robert Weissert, Fabienne Brilot

**Affiliations:** ^1^Department of Neurology, University of Regensburg, Regensburg, Germany; ^2^Brain Autoimmunity Group, Institute for Neuroscience and Muscle Research, Kids Research Institute at the Children’s Hospital at Westmead, Brain and Mind Centre, University of Sydney, Sydney, NSW, Australia

**Keywords:** multiple sclerosis, neuromyelitis optica spectrum disorder, autoimmune encephalitis, T cell, B cell, NMDA receptor, myelin oligodendrocyte glycoprotein, aquaporin-4

**Editorial on the Research Topic**

**Induction of Central Nervous System Disease by the Adaptive Immune Response**

T and B cells are of paramount importance in autoimmune diseases. This has been recognized for a long time and is not called into question. Nevertheless, it is often surprising how much energy it takes to convince colleagues that are not directly working in the field of neuroimmunology that the role of the adaptive immune responses is getting more and more important in many diseases of the central nervous system (CNS). In the past, many of those have not been considered to have an autoimmune origin, namely diseases with behavioral and/or psychiatric phenotypes. A prototype example of such a novel type of disease is anti-N-methyl d-aspartate receptor (anti-NMDAR) encephalitis which often mimics in certain aspects psychiatric diseases, such as schizophrenia or depression (1). In this specific form of autoimmune encephalitis (AE) that can have a paraneoplastic or purely autoimmune origin, antibodies target the NMDAR that is subsequently being internalized (2). This leads to changes in neural functioning with consequences on behavior. So far, the B cell and antibody side of this disease has been investigated in much detail, there is still incomplete knowledge regarding the T cell response. More and more CNS antigens exposed on cells are presently recognized as autoantigens of autoimmune CNS disorders. Based on this scientific progress, we decided that it would be of interest to summarize the current state of the field of autoimmune CNS disorders in which the adaptive immunity is the major disease driver.

Ehrenreich summarizes in her article the current understanding of anti-NMDAR encephalitis. She stresses the importance of the presence of anti-NMDAR antibodies in cerebrospinal fluid for disease precipitation (3). She also provides recommendations for the clinical diagnosis of this novel disease entity. Platt et al. also focus on the role of antibodies in various types of AE and discuss the triggers and induction of CNS autoimmune diseases. Strong emphasis is put on the blood–brain barrier and its role in AE. Zong et al. assess the impact of autoantibodies against additional target structures and consequences on disease phenotypes such as emergence of depression. The authors stress the value of broader assessment of antibody responses against different targets such as receptor complex, as well as and discrete epitopes within targets.

Pilli et al. review the current knowledge regarding T and B cell immunity against various antigens expressed in the CNS in multiple sclerosis (MS), neuromyelitis optica spectrum disorders (NMOSDs), and AE and assessment of immune responses to such CNS antigens using various methodological approaches.

Weissert summarizes the current knowledge regarding presence and localization of such antigens in CNS target cells and the distribution of such antigens within the cell. Differences and similarities between autoimmune and paraneoplastic immune-mediated disorders of the CNS are also discussed. The current understanding of the impact of the major histocompatibility complex (human leukocyte antigens) in MS as well as in NMOSD and AE is presented. Parnell and Booth review the current knowledge regarding genetic regulation in MS. They provide insight into the genetic regulation of function of single cell types by multiple genes, an interesting research area in the context of recent progresses in immunogenetics.

NMOSDs have been in the focus of research for the last years since the discovery of immunity against aquaporin-4, a water channel protein, expressed on astrocytes (4). Long et al. provide novel data that indicates that the disease course in NMOSD can strongly vary dependent on the initial symptom of the presentation of the disease. Zhao et al. provide some additional insight about the strong therapeutic effects of rituximab in patients with NMOSD. They found a reduction of circulating T follicular helper (cTfh) cells after treatment with rituximab in NMOSD patients. They conclude that this reduction in cTfh cell numbers and the reduced function of such cells could contribute to the observed beneficial effects of treatment of rituximab in NMOSD patients. This data could also be of relevance for other autoimmune disease of the CNS like AE.

Myelin oligodendrocyte glycoprotein (MOG) is a protein which is exposed on the outer surface of the myelin sheath. Research regarding this molecule in relation to MS has started in the 80s of the last century (5). Presently, the role of MOG in the pathogenesis of MS is still strongly debated. A subgroup of patients with NMOSD has persistent immunity against MOG (6). Peschl et al. reviewed in their article the current knowledge regarding MOG-directed autoimmunity in humans and experimental models of MS. The knowledge on MOG immunity that has been consolidated over the last decades will be of value also for other autoimmune diseases of the CNS.

Greer et al. demonstrate reactivity in patients with MS against novel thyroid-derived autoantigens. They also show overlapping autoimmunity against CNS and thyroid antigens in patients. These data are possibly of great importance and can explain much of the observed comorbidity of CNS demyelination in MS and thyroid disease. Interestingly, all the affected individuals were females underscoring the impact of genetics or/and hormonal regulation.

Autophagy has a strong impact on development of autoimmunity (7) and is a very important topic that is relevant for the pathogenesis of MS, NMOSD, and AE is. The field is currently fast-growing and much will be learned in the future about the relevance of autophagy in CNS-related autoimmune disorders. Keller and Lünemann et al. provide a current overview about autophagy and potential impact on immune-mediated diseases of the CNS.

Beside the ambitious research themes and stringent data that have been summarized in the collection of articles, there is another great serendipitous achievement. The researchers that contributed articles work in research institutions all over the world namely in Europe, Asia, USA, and Australia. Most of them did not previously know each other but were enthusiastic to be part of this project based on shared knowledge. Possibly this has already lead to novel research projects, collaborations, and funding. Finally, we want to thank the Frontiers Multiple Sclerosis and Neuroimmunology team for their continuing support, and especially the many colleagues who served as critical referees and contributed to the all-important peer-review process of this research topic.

## Author Contributions

RW and FB drafted and wrote the Editorial.

## Conflict of Interest Statement

The authors declare that the research was conducted in the absence of any commercial or financial relationships that could be construed as a potential conflict of interest.
